# GSK-3α/β Activity Negatively Regulates MMP-1/9 Expression to Suppress *Mycobacterium tuberculosis* Infection

**DOI:** 10.3389/fimmu.2021.752466

**Published:** 2022-01-12

**Authors:** Xinying Zhou, Linmiao Lie, Yao Liang, Hui Xu, Bo Zhu, Yingqi Huang, Lijie Zhang, Zelin Zhang, Qianna Li, Qi Wang, Zhenyu Han, Yulan Huang, Honglin Liu, Shengfeng Hu, Chaoying Zhou, Qian Wen, Li Ma

**Affiliations:** Institute of Molecular Immunology, School of Laboratory Medicine and Biotechnology, Southern Medical University, Guangzhou, China

**Keywords:** *Mycobacteria tuberculosis*, macrophages, GSK-3α/β, MMP-1, MMP-9

## Abstract

Tuberculosis (TB) caused by *Mycobacterium tuberculosis* (Mtb) infection is the deadliest infectious disease and a global health problem. Macrophages (Mφs) and neutrophils that can phagocytose Mtb represent the first line of immune response to infection. Glycogen synthase kinase-3α/β (GSK-3α/β) represents a regulatory switch in host immune responses. However, the efficacy and molecular mechanisms of how GSK-3α/β interacts with Mtb infection in Mφs remain undefined. Here, we demonstrated that Mtb infection downregulated GSK-3α/β activity and promoted matrix metalloproteinase-1 (MMP-1) and MMP-9 expressions in Mφs derived from acute monocytic human leukemia THP-1 cells (THP-1-Mφs). We confirmed the upregulation of MMP-9 expression in tissues of TB patients compared with patients of chronic inflammation (CI). In THP-1-Mφs and C57BL/6 mice, GSK-3α/β inhibitor SB216763 significantly increased MMP-1/9 production and facilitated Mtb load, while MMP inhibitors blocked MMP-1/9 expression and Mtb infection. Consistently, GSK-3α/β silencing significantly increased MMP-1/9 expression and Mtb infection, while overexpression of GSK-3α/β and constitutive activated GSK-3α/β mutants significantly reduced MMP-1/9 expression and Mtb infection in THP-1-Mφs. MMP-1/9 silencing reduced Mtb infection, while overexpression of MMP-1/9 promoted Mtb infection in THP-1-Mφs. We further found that GSK-3α/β inhibition increased Mtb infection and MMP-1/9 expression was blocked by ERK1/2 inhibitor. Additionally, we showed that protein kinase C-δ (PKC-δ) and mammalian target of rapamycin (mTOR) reduced GSK-3α/β activity and promoted MMP-1/9 production in Mtb-infected THP-1-Mφs. In conclusion, this study suggests that PKC-δ-mTOR axis suppresses GSK-3α/β activation with acceleration of MMP-1/9 expression through phospho-ERK1/2. These results reveal a novel immune escape mechanism of Mtb and a novel crosstalk between these critical signaling pathways in anti-TB immunity.

## Introduction

Tuberculosis (TB) caused by *Mycobacterium tuberculosis* (Mtb) infection remains a global health problem (1). TB pathogenesis is driven by a complex interplay between host immune system and survival strategies of the bacterium. Mtb replicates intracellularly, mainly within innate immune cells including macrophages (Mφs) and neutrophils, which act as the early immune responders against Mtb infection. The successful establishment of long-term Mtb infection rests upon its ability to convert Mφs into a permissive cellular niche to circumvent host immune response, which ultimately leads to the development of TB (2). Therefore, understanding of how Mtb infection regulates host factors may facilitate the development of novel targets for TB therapy.

Glycogen synthase kinase-3α/β (GSK-3α/β) is a multifunctional serine/threonine kinase capable of phosphorylating and inactivating glycogen synthase (GS) (3). Under basal cellular conditions, GSK-3α/β is a constitutively active serine/threonine kinase (3). Upon cellular stimuli, GSK-3α and GSK-3β can be phosphorylated on Serine 21 and Serine 9, resulting in loss of kinase activity. GSK-3α/β plays vital roles in host immune responses through regulating different signaling pathways of immune cells (3, 4). Therefore, the dysregulation of GSK-3α/β has been linked to diverse diseases, including infectious disease, cancer, Alzheimer’s disease, bipolar disorder, and diabetes (3). In infectious disease, on one hand, GSK-3α/β activation can be detrimental to the host response against *Francisella tularensis* LVS infection by restraining inflammatory cytokine response (5). On the other hand, GSK-3α/β can be beneficial to the host immune response against virus infection through activating IRF3 and NF-κB signaling pathways and IFN-β induction (6). It is reported that Mtb tyrosine phosphatase PtpA led to dephosphorylation of GSK-3α (7). In human primary dendritic cells (DC), Mtb infection modulated pro- and anti-inflammatory cytokine production through mTOR/GSK-3β axis (8). However, the exact efficacy and molecular mechanisms as how GSK-3α/β interacts with Mtb infection in Mφs remain undefined.

Enzymes of matrix metalloproteinases (MMPs) family play key roles in host immune response against TB (9). Among numerous MMPs secreted from monocytes and macrophages infected with Mtb, MMP-1, MMP-2, MMP-3, MMP-8, and MMP-9 are most extensively studied isotypes with critical roles in host defense against Mtb infection (10). In our study, only expression of MMP-1 and MMP-9 was regulated by GSK-3α/β in Mφs. Most of studies pay close attention to collagen breakdown and alveolar destruction mediated by MMP-1 production from Mφs, and initiation of recruitment of new monocytes to develop granuloma mediated by MMP-9 production (11). MMP-1/9 expression can be regulated by multiple signaling pathways. Specifically, extracellular signal-regulated kinase 1/2 (ERK1/2) has been identified in charge of MMP expression, and the phosphorylation status of ERK1/2 is proposed being regulated by GSK-3 in several diseases (12–16). This raised our curiosity as to how GSK-3α/β interacts with MMP-1/9 and ERK1/2 in combating Mtb infection in Mφs.

Evidence indicates that GSK-3α/β represents a point of convergence of different signal transduction pathways of the immune system (3). Inhibition of mammalian target of rapamycin (mTOR) increased GSK-3β activity to regulate pro- and anti-inflammatory cytokine production in LPS-stimulated Mφs (17). Protein kinase C (PKC) has been identified as a key regulatory factor to stimulate mTOR activation (18). PKC activation can also lead to reduction of GSK-3β activity independent of mTOR to control lysosome (19). Thus, it is of an intriguing question whether PKC-mTOR axis plays a crucial role in regulating GSK-3α/β activity and anti-TB immunity. In this study, we show that Mtb infection downregulates GSK-3α/β activity and promotes MMP-1/9 expression in human Mφs. Further investigation identifies that GSK-3α/β combats Mtb infection through MMP-1/9 production, which is regulated by upstream of PKC-mTOR axis and ERK1/2 phosphorylation. These results revealed a novel immune escape mechanism of Mtb in Mφs and a novel crosstalk mechanism between these critical signaling pathways in anti-TB immunity.

## Materials and Methods

### Ethics Approval

This study was approved by the Ethics Committee of Southern Medical University with written informed consent from all subjects. All patients with active pulmonary TB (PTB), lymphatic TB, and chronic inflammation (CI) enrolled in this study have written informed consent. The protocol was approved by the ethics committee of the Southern Medical University. The animal ethical certification and animal handling procedures were approved by the Animal Experimental Center in Southern Medical University. Highly pathogenic microorganism laboratory management commitment letter was approved by Southern Medical University.

### Patients

Patients with active PTB, lymphatic TB, and CI were diagnosed in and recruited from the Guangzhou Chest Hospital (Guangzhou, Guangdong, China). We collected six patients with active PTB (4 males and 2 females), six patients with lymphatic TB (2 males and 4 females), and eleven patients with CI (7 nales and 4 females).

### Cell Culture

Human acute monocytic leukemia cells (THP-1) were purchased from CELLCOOK (CC1904, Guangzhou, China) and cultured in RPMI-1640 medium (Corning, NY, USA) containing 10% heat-inactivated FBS in 5% CO_2_ cell culture incubator at 37°C. THP-1 cells were stimulated with 100 ng/ml phorbol-12-myristate-13-acetate (PMA) (Pepro Tech, NJ, USA) for 48 h to turn into mature Mφs (THP-1-Mφs). THP-1-Mφs were maintained in complete RPMI-1640 medium without PMA treatment for 24 h for further experiments.

### Mycobacteria Culture and Infection

Highly pathogenic microorganism laboratory management commitment letter was approved by Southern Medical University. All Mtb infection experiments have been operated in BSL-3 lab of Southern Medical University. Mtb strain H37Rv (American Type Culture Collection) was cultured in 7H9 broth (Becton Dickinson, New Jersey, USA) with 10% OADC (0.06% (v/v) oleic acid (SIGMA, St. Louis, MO, USA), 5% albumin (SIGMA, St. Louis, MO, USA), 100 mM glucose (GHTECH, Guangzhou, China), 0.003% catalase (SIGMA, St. Louis, MO, USA), and 145 mM NaCl (GHTECH, Guangzhou, China) at 37°C with 5% CO_2_. Grinded the clumps of bacteria into bacterium suspension and measured the concentration of bacteria at OD_600_ absorbance. Mtb at multiplicity of infection (MOI) of 5 has been applied to infect THP-1-Mφs for colony-forming assay (CFU), and MOI of 2 has been used to infect THP-1-Mφs for other experiments. Mtb infected mice are kept in biosafety cabinet. The experimental treatment or anatomical operation was carried out on the negative pressure ultra clean table. The infected animals were carried out in the isolation cover and placed on the plate. The experimental area of infected animals is equipped with an electric steam autoclave to disinfect and sterilize various items. Mice excreta, bedding and residual feed can only be discarded after thorough disinfection and sterilization. After the sacrifice, the mice were sent to animal center of Southern medical university and sterilized by high-pressure disinfection and then sealed and packaged for incineration. Cages and experimental equipment were cleaned after high-pressure sterilization.

### Treatment of Reagents in Mφs

THP-1-Mφs were pretreated with specific inhibitor of SB216763 for GSK-3α/β (20 µM) (Selleck, Houston, USA), BB94 for MMPs (20 µM) (Selleck, Houston, USA), SB3CT for MMP-9 (Selleck, Houston, USA), GO6983 for PKCs (1 µM) (Selleck, Houston, USA), rapamycin for mTOR (1 µM) (Selleck, Houston, USA), U0126 for ERK (10 µM) (Selleck, Houston, USA), activator of PMA for PKCs (20 µM) (Selleck, Houston, USA), and dimethylsulfoxide (DMSO) as solvent control for 2 h before Mtb infection for 24 or 48 h.

### Cell Viability

The Trans Detect Cell Counting Kit-8 (CCK-8) (TransGene Biotech, Beijing, China) was based on the conversion of a water-soluble tetrazolium salt, and 2-(2-methoxy-4-nitrophenyl)-3-(4-nitrophenyl)-5-(2,4-disulfophenyl)-2H-tetrazolium, monosodium salt (WST-8) was used to detect cytotoxicity of THP-1-Mφs. Cells were cultured in 96-well plates at density of 1 × 10^5^ cells/ml for 24 and 48 h. The culture medium was replaced with fresh complete medium containing 10% CCK-8 solution, followed by further incubation for 1–4 h at 37°C. The Varioskan Flash (Thermo Fisher Scientific, Carlsbad, CA, USA) was used to measure absorbance at 450 and 630 nm within 30 min. The relative cell viability was calculated as a percentage of control values (blank).

### Small Interfering RNA Transfection and Transfection

Transient small interfering RNA (siRNA) targeting GSK-3α (NM_019884.2), GSK-3β (NM_001146156.2), PKC-α (NM_002737.3), PKC-β (NM_002738.7), PKC-γ (NM_002739.5), PKC-δ (NM_001354680.2), PKC-ϵ (NM_005400.3), MMP-1 (NM_002421.4), MMP-3 (NC_002422.5), MMP-9 (NM_004994.3) and si-NC (negative control) were synthesized by RiboBio according to NCBI gene database. The siRNA target sequences involved in this study were as follows: GSK-3α (1: GAACCCAGCTGCCTAACAA; 2: GATTGGCAATGGCTCATTT; 3: CAAGTTCCCTCAGATTAAA), GSK-3β (1: GGAAGCTTGTGCACATTCA; 2: GGACTATGTTCCGGAAACA; 3: GGACCCAAATGTCAAACTA), MMP-1 (1: GCTTGAAGCTGCTTACGAA; 2: GGACCATGCCATTGAGAAA; 3: GCACATGACTTTCCTGGAA), MMP-3 (1: GAGAAATCCTGATCTTTAA; 2: GCAAGGACCTCGTTTTCAT; 3: GCCAGGGATTAATGGAGAT), MMP-9 (1: GTACCGCTATGGTTACACT; 2: GGTTCCAACTCGGTTTGGA; 3: GCAACGTGAACATCTTCGA), PKC-α (1: GCACAACGTTTCCTATCCA; 2: GAAGGGTTCTCGTATGTCA; 3: GGACTGGGATCGAACAACA), PKC-β (1: GAAGGACGTTGTGATCCAA; 2: GGATGAAACTGACCGATTT; 3: GCTGCTTTGTGGTGCACAA), PKC-δ (1: GCTTCAAGGTTCACAACTA; 2: GCAAGTGCAACATCAACAA), PKC-γ (1: CTCGGAACCTGACGAAACA; 2: CATCGACGATGCCACGAAT; 3: CCCGTAACCTAATTCCTAT), PKC-ϵ (1: GACGTGGACTGCACAATGA; 2: GAGTGTATGTGATCATCGA; 3: GGGCAAAGATGAAGTATAT). Lipofectamine 2000 (Thermo Fisher Scientific, Carlsbad, CA, USA) and optiMEM (Gibco, Life Technologies, NY, USA) as transfection reagent were used to mix with siRNAs at final concentration of 100 nM. The mixed transfection reagents were incubated at room temperature for 20 min and added dropwise into THP-1-Mφs containing RPMI-1640 medium. After incubation for 4–6 h, the culture medium was replaced with fresh complete medium and cells were incubated for another 48 h for further experiments.

### Lentiviral-Mediated Overexpression

The X-tremeGENE HP DNA transfection reagent (Roche, Basel, Switzerland) was used and operated according to the manufacturer’s instruction. For GSK-3α/β overexpression, pSLenti-SFH-EGFP-P2A-Puro-CMV-MCS-3xFLAG-WPRE vector (OBiO Technology, Shanghai, China) was used as empty lentivirus, pSLenti-SFH-EGFP-P2A-Puro-CMV-GSK3A(S21A)/GSK3B(S9A)-3xFLAG-WPRE were used for GSK-3α/β and GSK-3αS21A/GSK-3βS9A overexpression. For MMP-1, MMP-2, and MMP-9 overexpression, pLVX-Puro vector of MMP-1 (NM_002421.4), MMP-2 (NM_004994.3), and MMP-9 (NM_004994.3) with C-terminal Flag tag were used (OBiO Technology, Shanghai, China). Third-generation lentiviral packaging system with helper plasmids of pLP1, pLP2, and pLP/VSVG were applied. Lentiviral pseudoparticles were generated in HEK293T cells, and virus-containing medium was collected after 72 h posttransfection and concentrated with Lenti-Concentin Virus Precipitation Solution 5× (exCELL EMB810A-1, Beijing, China). Lentiviral pseudoparticles were stored at −80°C for further experiments. THP-1 cell lines were cultured in 6-well plates at a density of 5 × 10^5^ cells per well and transduced with lentiviral pseudoparticles at 37°C for 72 h. After culturing the passaging cells for 7 days, we collected all the cells by flow cytometry (BD Biosciences, San Jose, CA, USA) to select the green fluorescent protein (GFP)-marked positive ones. GFP-marked THP-1 cells transfected with empty lentivirus has been used as control cells.

### RNA Extraction and Quantitative Real-Time PCR

RNA of macrophages lysed with TRIzol (Thermo Fisher Scientific, Carlsbad, CA, USA) was quantified by Nanodrop 2000c (Thermo Fisher Scientific, Carlsbad, CA, USA) and reverse transcribed into the same amount of cDNA by the TransScript One-Step gDNA Removal and cDNA Synthesis SuperMix kit (TransGen Biotech, China). mRNA level of individual gene was quantified by quantitative real-time PCR (qRT-PCR) using a SYBR^®^ Premix Ex TaqTM II (Tli RNaseH Plus) (TaKaRa, Beijing, China) on a LightCycler 480 thermocycler (Roche, Basel, Switzerland). After initial denaturation at 95°C for 2 min, targeted genes were amplified and quantitated (95°C for 15 s, 60°C for 15 s) for 45 cycles, followed by a final extension at 68°C for 20 s. After normalizing all PCR products with respect to GAPDH transcript and using the 2^−△△CT^ method to calculate, the expression level of individual genes can be expressed as fold change. The complete primers are GAPDH (F: GTCTCCTCTGACTTCAACAGCG, R: ACCACCCTGTTGCTGTAGCCAA); TNF-α: (F: CTCTTCTGCCTGCTGCACTTTG, R: ATGGGCTACAGGCTTGTCACTC); IL-1β (F: CCACAGACCTTCCAGGAGAATG, R: GTGCAGTTCAGTGATCGTACAGG); IL-6 (F: AGACAGCCACTCACCTCTTCAG, R: TTCTGCCAGTGCCTCTTTGCTG); IL-10 (F: TCTCCGAGATGCCTTCAGCAGA, R: TCAGACAAGGCTTGGCAACCCA); IFN-α (F: TGGGCTGTGATCTGCCTCAAAC, R: CAGCCTTTTGGAACTGGTTGCC); IFN-β (F: CTTGGATTCCTACAAAGAAGCAGC, R: TCCTCCTTCTGGAACTGCTGCA); IFN-γ (F: GAGTGTGGAGACCATCAAGGAAG, R: TGCTTTGCGTTGGACATTCAAGTC); IRF1 (F: GAGGAGGTGAAAGACCAGAGCA, R: TAGCATCTCGGCTGGACTTCGA); Mx1 (F: GGCTGTTTACCAGACTCCGACA, R: CACAAAGCCTGGCAGCTCTCTA); Rsad2 (F: CCAGTGCAACTACAAATGCGGC, R: CGGTCTTGAAGAAATGGCTCTCC); ISG15 (F: CTCTGAGCATCCTGGTGAGGAA, R: AAGGTCAGCCAGAACAGGTCGT); MMP-1 (F: ATGAAGCAGCCCAGATGTGGAG, R: TGGTCCACATCTGCTCTTGGCA); MMP-2 (F: AGCGAGTGGATGCCGCCTTTAA, R: CATTCCAGGCATCTGCGATGAG); MMP-3 (F: CACTCACAGACCTGACTCGGTT, R: AAGCAGGATCACAGTTGGCTGG); MMP-8 (F: CAACCTACTGGACCAAGCACAC, R: TGTAGCTGAGGATGCCTTCTCC); MMP-9 (F: GCCACTACTGTGCCTTTGAGTC, R: CCCTCAGAGAATCGCCAGTACT); GSK-3α (F: GCAGATCATGCGTAAGCTGGAC, R: GGTACACTGTCTCGGGCACATA); GSK-3β (F: CCGACTAACACCACTGGAAGCT, R: AGGATGGTAGCCAGAGGTGGAT); PKC-α (F: GCCTATGGCGTCCTGTTGTATG, R: GAAACAGCCTCCTTGGACAAGG); PKC-β (F: GAGGGACACATCAAGATTGCCG, R: CACCAATCCACGGACTTCCCAT); PKC-γ (F: CCGCCTGTATTTCGTGATGGAG, R: CGATAGCGATTTCTGCCGCGTA); PKC-δ (F: GCTGACACTTGCCGCAGAGAAT, R: GCCTTTGTCCTGGATGTGGTAC); PKC-ϵ (F: AGCCTCGTTCACGGTTCTATGC, R: GCAGTGACCTTCTGCATCCAGA).

### Western Blot Analysis

After washing twice with PBS, cells were lysed with basic lysis buffer (composed of 455 mM Tris HCl (pH 6.8) (Sangon Biotech, Shanghai, China), 41.6 mM SDS (Zhuosheng Biotech, Shanghai, China), 26.9 μM bromophenol blue (Solarbio, Beijing, China), 30% (v/v) glycerol (SIGMA, St. Louis, MO, USA), and 10 μM dl-dithiothreitol (DTT) (SIGMA, St. Louis, MO, USA) to obtain total proteins. The proteins were denatured by heating at 100°C for 5 min, separated by SDS-PAGE and transferred to the PVDF membrane. Then the target protein membranes were cut out according to the precision plus protein TM standards (BIORAD, Hercules, CA, USA). Membranes were blocked with PBS-T (0.1% Tween-20 (GHTECH, Guangzhou, China) containing 5% (w/v) BSA (SIGMA, St. Louis, MO, USA) at room temperature for 1 h and inoculated with primary antibodies at 4°C for 16 h with gentle shaking. After washing for three times with PBS-T, the membranes were incubated with HRP-conjugated goat antirabbit or goat antimouse secondary antibodies (Cell Signaling Technology, Danvers, MA, USA) at room temperature for 1 h and washed for another three times with PBS-T. The separated protein band was visualized using Immobilon Western Chemiluminescence HRP substrate (ECL; Thermo Fisher Scientific, USA). Each measurement was performed in triplicate with similar results, and one representative result was shown. The integrated density of blotting bands was quantitatively analyzed by Image J software (National Institutes of Health, Bethesda, MD, USA) and normalized to 1.0 with GAPDH as a control. The antibodies used in this study are listed in **Supplementary Table S1**.

### Enzyme-Linked Immunosorbent Assay

Supernatants were harvested from the infected cells and mouse tissues centrifuged at 12,000 × g for 10 min to remove cell debris after grinding. Samples were assayed using enzyme-linked immunosorbent assay (ELISA) kits for MMP-1 (MultiSciences, Hangzhou, China) and MMP-9 (MultiSciences, Hangzhou, China). All procedures were performed according to the manufacturer’s instructions. The ELISA kits were equilibrated to room temperature in advance, and after dissolving and diluting the standard according to the standard curve, the sample supernatant was diluted to appropriate multiple dilutions. Diluted sample supernatant at 100 μl and the standard solution at different concentrations were added to the corresponding wells. Antibody solution (50 μl, 1:100) was then added to each well and all the samples were incubated at room temperature for 2 h. After washing for six times with washing buffer, enzyme solution (100 μl, 1:100) was added to each well and incubated at room temperature for 45 min. After washing repeatedly for six times, chromogenic substrate (100 μl) was added to all wells and incubated for 5–15 min avoiding light. Finally, 100 μl stop solution was added to stop the reaction. We measured the absorbance at 450 and 630 nm within 30 min by a Varioskan Flash (Thermo Fisher Scientific, Carlsbad, CA, USA). The concentration of the samples was calculated according to the standard curve and the OD value of the samples.

### Immunohistochemistry Assay

Archived human lymph node and lung tissues were fixed in 10% formalin, embedded in paraffin wax and deparaffinized in xylene. For immunohistochemical staining, parraffin sections (4 µm) were heated with 0.01 M citric acid of pH 6.0 for 3 min for antigen retrieval. The slices were treated with 3% H_2_O_2_ for 10 min to react with endogenous peroxidase. Sections were incubated with MMP-9 (1:500, Abcam Cambridge, UK) Ab overnight and secondary biotinylated anti-mouse (1: 50) followed by DAB substrate (UniCureLab). The slices were counterstained by hematoxylin and dehydrated by gradient alcohol. For slices of lungs, because the cells are relatively scattered and few, immunohistochemical staining for MMP-9 was quantified by positive cell rate analysis by using Image Pro Plus 6.0 software (Media Cybernetics, Inc., Rockville, MD, USA). We analyzed the number of positive cells with the same brown-yellow cell nucleus and the total number of cells in each photo, then the percentage of positive cells was calculated. Positive rate (%) is equal to the number of positive cells divided with the total number of cells multiplied with 100. For slices of lymph nodes, because the density and quantity of cells are relatively large, immunohistochemical staining for MMP-9 was quantified by area density analysis by using Image Pro Plus 6.0 software (Media Cybernetics, Inc., Rockville, MD, USA). The accumulated optical density (IOD) and the area of tissue pixels (area) of each photo are obtained. The area density = IOD/area. Higher area density is representative of the higher positive expression level. The antibodies used in this research are listed in **Supplementary Table S1**.

### Colony-Forming Unit Assay

THP-1-Mφs were infected with H37Rv of MOI = 5 for 1 h at 37°C (5% CO_2_). Cells were then washed with PBS for three times to remove extracellular bacteria, and complete medium was added to culture at different time points. Cells were lysed in ddH_2_O containing 0.01% Triton X-100. After serial dilution, 50 µl bacteria solution (1:1,000) was evenly spread on the 7H10 agar plate (Becton Dickinson, New Jersey, USA) and then cultured for 21–28 days at 37°C (5% CO_2_). To detect the direct effect of inhibitors on Mtb infection, H37Rv of 1 × 10^6^ was incubated with various inhibitors for 2 h. Mtb suspension was then washed with PBS for three times to remove inhibitors, and complete medium was added to culture at 48 h. A total of 50 µl bacterial solution (1:1,000) was evenly spread on the 7H10 agar plate (Becton Dickinson, New Jersey, USA) and then cultured for 21–28 days at 37°C (5% CO_2_).

### Animal Treatment

Wild-type female C57BL/6 mice (6 weeks old) were obtained from the Laboratory Animal Center of Southern Medical University. Mice were randomly divided into four groups (*n* = 4/group) as follows: (1) intraperitoneal saline + DMSO, (2) intraperitoneal saline + SB216763 (20 mg/kg) (Selleck, Houston, TX, USA) dissolved in DMSO, (3) intraperitoneal saline + SB3CT (20 mg/kg) (Selleck, Houston, TX, USA) dissolved in DMSO, and (4) intraperitoneal saline + SB3CT (20 mg/kg) + SB216763 (20 mg/kg) dissolved in DMSO. To establish a pulmonary tuberculosis model, mice were injected H37Rv intraperitoneally with 1 × 10^6^ CFU/mouse on day 0. SB216763-treated mice were administered with saline + SB216763 dissolved in DMSO intravenously at day 0 and then intraperitoneally twice a week until day 28. SB3CT-treated mice were intraperitoneally injected with saline + SB3CT dissolved in DMSO daily until day 28. The control group was injected with saline + DMSO as vehicle control. The mice were sacrificed on days 7 and 28, and lung and spleen tissues were grinded for CFU assay. The supernatant of lung and spleen tissue grinding was used to detect the expression of MMP-9 by ELISA assay.

### Statistical Analysis

Statistical analysis was performed with Graphpad Prism 5.0 Software. Results are presented as means ± SD or means ± SEM of at least three independent experiments. Statistical analysis was performed using unpaired Mann-Whitney *U* test. ^*^ *p* ≤ 0.05 and ^**^ *p* ≤ 0.01 were considered statistically significant.

## Results

### GSK-3α/β Suppresses Mtb Infection in Mφs and in Mice

**Figure 1:**
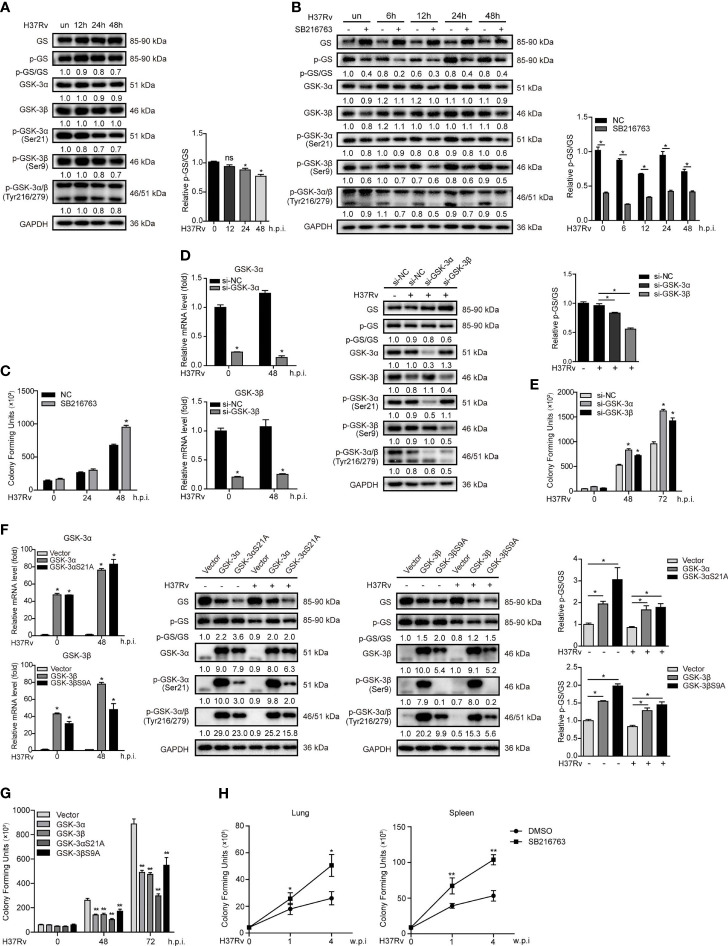
GSK-3α/β suppresses Mtb infection in Mφs and in mice. **(A)** Expressions of GSK-3α/β, phospho-GSK-3α/β, GS, and phospho-GS were detected in THP-1-Mφs upon Mtb infection at indicated time points by Western blot analysis. The ratio of expression of phosphorylated GS to total GS is shown in graph. **(B)** Expressions of GSK-3α/β, phospho-GSK-3α/β, GS, and phospho-GS were detected in THP-1-Mφs with pretreatment of 20 μM SB216763 for 2 h upon Mtb infection at indicated time points by Western blot analysis. The ratio of expression of phosphorylated GS to total GS is shown in graph. **(C)** The intracellular mycobacteria load was detected in THP-1-Mφs with pretreatment of SB216763 at 24 and 48 h.p.i. of Mtb by CFU assay (means ± SD, *n* = 3 independent experiments with each 4 replicates). **(D)** GSK-3α- and GSK-3β-silenced THP-1-Mφs were detected for GSK-3α/β mRNA expression by qRT-PCR, and expressions of GSK-3α/β, phospho-GSK-3α/β, GS, and phospho-GS by Western blot analysis upon 48 h of Mtb infection (means ± SD, *n* = 3 independent experiments with each 4 replicates). The ratio of expression of phosphorylated GS to total GS is shown in graph. **(E)** The intracellular Mtb load was determined by CFU analysis in GSK-3α/β-silenced THP-1-Mφs at 48 and 72 h.p.i. of Mtb (means ± SD, *n* = 3 independent experiments with each 4 replicates). **(F)** THP-1-Mφs overexpressing GSK-3α, GSK-3β, GSK-3αS21A, and GSK-3βS9A were detected for GSK-3α/β mRNA expression by qRT-PCR, and expressions of GSK-3α/β, phospho-GSK-3α/β, GS, and phospho-GS by Western blot analysis upon 48 h of Mtb infection (means ± SD, *n* = 3 independent experiments with each 4 replicates). The ratio of expression of phosphorylated GS to total GS is shown in graph. **(G)** The intracellular bacteria load was detected by CFU analysis in THP-1-Mφs overexpressing GSK-3α, GSK-3β, GSK-3αS21A, and GSK-3βS21A at 48 and 72 h.p.i. of Mtb (means ± SD, *n* = 3 independent experiments with each 4 replicates). **(H)** Mice infected of H37Rv were treated with SB216763 (20 mg/kg, *n* = 5) or DMSO (*n* = 5), and the bacterial load of lungs and spleens were detected by CFU analysis at 1 and 4 weeks postinfection (means ± SD, *n* = 3 independent experiments with each 4 replicates). For Western blot assay, GAPDH served as internal control. Data presented are from one of at least three independent experiments with similar results. The numbers below immunoblot correspond to band-integrated density ration of target protein to GAPDH. ^*^ *p* ≤ 0.05 and ^**^ *p* ≤ 0.01 were considered statistically significant. SB216763: GSK-3α/β inhibitor.

To investigate the role of GSK-3α/β activity in TB, we first detected the expression of phosphorylated GSK-3α/β (Ser21/9 and Tyr216/279) and phosphorylated GS in THP-1-Mφs upon Mtb infection by Western blot assay. The results showed that both Ser21/9 and Tyr216/279 phosphorylation of GSK-3α/β were significantly decreased, making it difficult to estimate how GSK-3α/β activity is affected by Mtb infection. We then found that Mtb infection decreased the ratio of phosphorylation level (activated form) to total protein expression of GS, indicating GSK-3α/β activity was inhibited by Mtb infection (**Figure 1A**). Next, GSK-3α/β inhibitor SB216763 was used to evaluate the effect of GSK-3α/β on Mtb infection. SB216763 suppressed GSK-3α/β activity indicated by the decreased ratio of activated GS to total GS upon Mtb infection at different time points (**Figure 1B**). CFU assay showed that SB216763 significantly increased Mtb infection in THP-1-Mφs upon 48 h of infection (**Figure 1C**). These results were found not related to enhancement of cell proliferation with SB216763 treatment by CCK-8 assay (**Supplementary Figure S1A**) or direct increase of Mtb infection (**Supplementary Figure 1B**). Two siRNAs targeting GSK-3α and GSK-3β were used to knock down GSK-3α and GSK-3β. As expected, GSK-3α and GSK-3β silencing decreased mRNA and protein expression without off-target effects between each other, and also decreased GSK-3α/β activity indicated by the ratio of activated GS to total GS upon 48 h of Mtb infection (**Figure 1D**). Correspondingly, GSK-3α and GSK-3β silencing led to a significant increase of Mtb load upon 48 and 72 h Mtb infection (**Figure 1E**). Subsequently, we constructed THP-1-Mφs that stably expressed GSK-3α and GSK-3β, and constitutively active forms of GSK-3α/β (GSK-3αS21A, GSK-3βS9A) with lentiviral vectors. Overexpressing both wildtype and mutations of GSK-3α/β potently increased levels of mRNA, protein, and the ratio of activated GS to GS (**Figure 1F**) and significantly attenuated intracellular Mtb infection upon 48 and 72 h of infection (**Figure 1G**). These results were not due to the off-target effect of GSK-3α and GSK-3β overexpression because they did not interact with each other by detecting their protein expression respectively (**Supplementary Figure S1C**). Moreover, both wildtype and mutations of GSK-3α/β could inhibit Mtb infection induced by SB216763 treatment for 48 h (**Supplementary Figure S1D**).

We further investigate the effects of GSK-3α/β on Mtb infection in mice. CFU assay showed that SB216763 treatment led to significant increase of bacterial load in lungs and spleens of mice after 1 and 4 weeks of Mtb infection (**Figure 1H**). These data indicated that Mtb infection suppressed in GSK-3α/β plays an important role in Mtb eradication in macrophages and in mice.

### GSK-3α/β Inhibits MMP-1/9 Expression in Mtb-Infected Mφs

**Figure 2:**
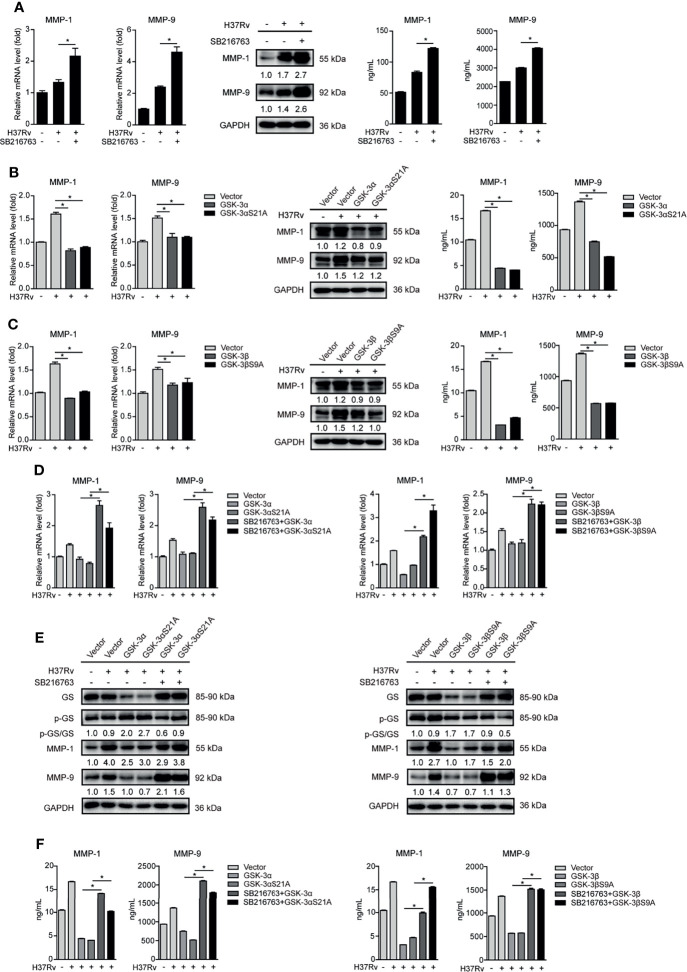
GSK-3α/β inhibits MMP-1/9 expression in Mtb-infected Mφs. **(A)** Intracellular mRNA, protein expression, and secretion of MMP-1/9 were detected in THP-1-Mφs pretreated with SB216763 for 2 h before 48 h of Mtb infection by qRT-PCR, Western blot, and ELISA analyses (means ± SD, *n* = 3 independent experiments with each 4 replicates). **(B, C)** Intracellular mRNA, protein expression, and secretion of MMP-1/9 were detected in THP-1-Mφs overexpressing GSK-3α, GSK-3αS21A, GSK-3β, and GSK-3βS9A with H37Rv infection for 48 h by qRT-PCR, Western blot, and ELISA analyses (means ± SD, *n* = 3 independent experiments with each 4 replicates). **(D–F)** SB216763 was used to treat THP-1-Mφs overexpressing GSK-3α, GSK-3αS21A, GSK-3β, and GSK-3βS9A upon 48 h of Mtb infection, and intracellular mRNA, protein expression, and secretion of MMP-1/9 were detected by qRT-PCR, Western blot, and ELISA analyses (means ± SD, *n* = 3 independent experiments with each 4 replicates). For Western blot assay, GAPDH served as internal control. Data presented are from one of at least three independent experiments with similar results. The numbers below immunoblot correspond to band-integrated density ration of target protein to GAPDH. ^*^ *p* ≤ 0.05 was considered statistically significant. SB216763: GSK-3α/β inhibitor.

Autophagy, proinflammatory, and anti-inflammatory cytokines including interferons (IFNs) and IFN-stimulated genes (ISGs) are important host immune responses downstream of GSK-3α/β signaling (2). Upon SB216763 treatment, the conversion of LC3-I to LC3-II indicated by ratio of LC3-II/LC3-I expression was decreased at 8 and 12 hours postinfection (h.p.i.), and modestly increased at 48 h.p.i. Expression of p62 protein increased at 12, 24, and 48 h.p.i. (**Supplementary Figure S2A**). These results indicated that GSK-3α/β activity irregularly regulated autophagy. Expression of IL-6, TNF-α, IL-1β, and IL-10 mRNA was detected in Mtb-infected THP-1-Mφs at 4, 8, 12, 24, and 48 h.p.i. with SB216763 treatment. We found that expression of IL-6 and TNF-α significantly decreased at 48 h.p.i.; however, expression of IL-1β and IL-10 significantly increased at 24 and 48 h.p.i. These cytokines were irregularly regulated by SB216763 treatment (**Supplementary Figure S2B**). We further demonstrated that expression of IFN-α and IFN-γ, as well as Mx1, Rsad2, and ISG15, was not influenced by SB216763 treatment. IFN-β and IRF1 expressions were significantly decreased at 48 h.p.i. (**Supplementary Figure S2C**). Our previous study showed that IRF1 did not affect MMP expression (20). We speculated that GSK-3α/β suppressing Mtb infection might be independent of autophagy, inflammatory cytokine production, and IFN signaling. To further investigate how GSK-3α/β activity affects Mtb infection, MMPs including expression of MMP-1, MMP-2, MMP-3, MMP-8, and MMP-9 were analyzed. We found that Mtb infection significantly increased MMP-1, MMP-3, MMP-8, and MMP-9 but decreased MMP-2 mRNA expression with time (**Supplementary Figure S3A**). Next, we observed that mRNA expression of MMP-1, MMP-3, and MMP-9 was significantly increased by GSK-3α and GSK-3β silencing in THP-1-Mφs upon Mtb infection; however, MMP-3 protein expression was not influenced (**Supplementary Figures S3B, C**). Additionally, MMP-3 silencing did not affect Mtb infection in THP-1-Mφs (**Supplementary Figures S3D, E**). For this reason, we further investigate the molecular mechanism of how GSK-3α/β regulates MMP-1 and MMP-9 upon Mtb infection. We further observed that SB216763 treatment resulted in significant increase of intracellular mRNA and protein expression as well as extracellular supernatant production of MMP-1 and MMP-9 (**Figure 2A**). Inversely, GSK-3α/β overexpression (**Figure 2B**) and its constitutively active forms (GSK-3αS21A, GSK-3βS9A) (**Figure 2C**) significantly decreased intracellular expression and supernatant production of MMP-1 and MMP-9 in Mtb-infected THP-1-Mφs. Subsequently, we demonstrated that SB216763 treatment could decrease the ratio of activated GS to total GS caused by GSK-3α/β and GSK-3αS21A/GSK-3βS9A overexpression, while SB216763 treatment restored intracellular expression and supernatant production of MMP-1 and MMP-9 reduced by GSK-3α/β and GSK-3αS21A/GSK-3βS9A overexpression in THP-1-Mφs (**Figures 2D–F**). These results suggest that GSK-3α/β activation inhibits MMP-1/9 expression in Mtb infection in Mφs.

### GSK-3α/β Suppresses Mtb Infection Through Inhibiting MMP-1 and MMP-9 Expressions

**Figure 3:**
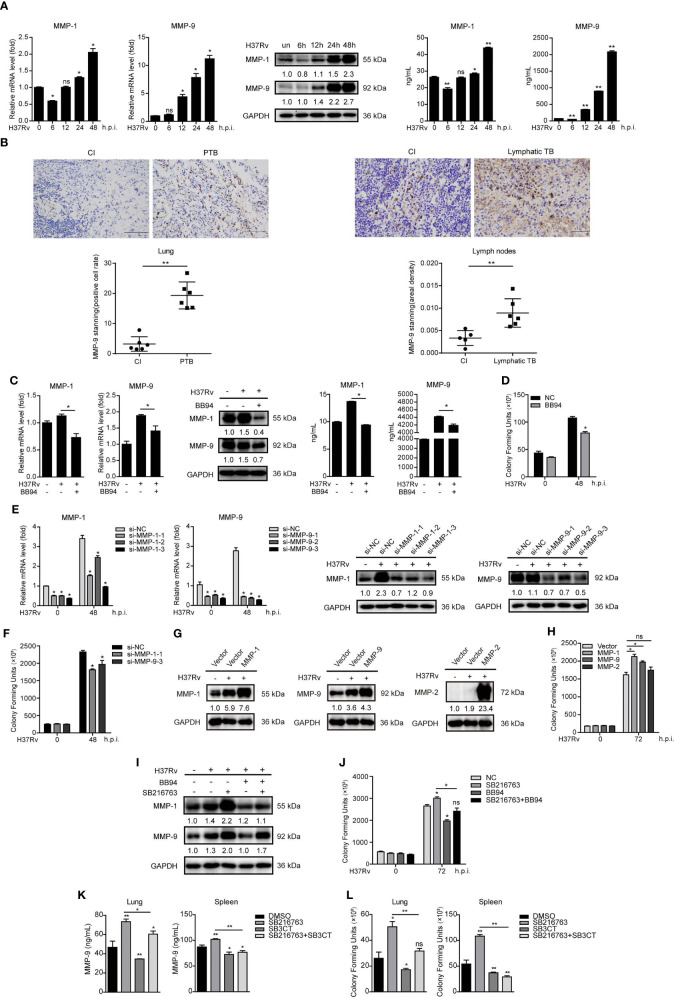
GSK-3α/β suppresses Mtb infection through inhibiting MMP-1/9 expression. **(A)** Intracellular mRNA, protein expression, and secretion of MMP-1/9 in THP-1-Mφs infected with Mtb at indicated time points were detected by qRT-PCR, Western blot, and ELISA analyses (means ± SD, *n* = 3 independent experiments with each 4 replicates). **(B)** Representative example of immunohistochemical staining for MMP-9 in lung biopsies of six patients with active pulmonary TB (PTB) and six patients with chronic inflammation (CI). Data are quantified by positive cell rate analysis (left). Scale bars, 50 μm. Representative example of immunohistochemical staining for MMP-9 in lymph node biopsies of six patients with lymphatic TB and five patients with chronic inflammation (CI). Data are quantified by area density analysis (right). Scale bars, 20 μm. **(C)** Intracellular mRNA, protein expression, and secretion of MMP-1/9 were analyzed in THP-1-Mφs pretreated with 20 μM BB94 for 2 h at 48 h.p.i. by qRT-PCR, Western blot, and ELISA analysis (means ± SD, *n* = 3 independent experiments with each 4 replicates). **(D)** CFU assay was applied to detect Mtb infection in THP-1-Mφs pretreated with 20 μM BB94 for 2 h at 48 h.p.i. (means ± SD, *n* = 3 independent experiments with each 4 replicates). **(E)** Intracellular mRNA and protein expression of MMP-1/9 were detected in MMP-1- and MMP-9-silenced THP-1-Mφs at 48 h.p.i. by qRT-PCR and Western blot analysis (means ± SD, *n* = 3 independent experiments with each 4 replicates). **(F)** CFU assay was applied to detect intracellular Mtb load in MMP-1 and MMP-9 silencing THP-1-Mφs at 48 h.p.i. (means ± SD, *n* = 3 independent experiments with each 4 replicates). **(G)** MMP-1, MMP-2, and MMP-9 expressions and **(H)** intracellular Mtb infection were detected in THP-1-Mφs with stable overexpression of MMP-1, MMP-2, and MMP-9 by qRT-PCR, Western blot analysis, and CFU assay (means ± SD, *n* = 3 independent experiments with each 4 replicates). **(I)** MMP-1/9 protein expression and **(J)** intracellular Mtb infection were detected in THP-1-Mφs pretreated with SB216763, BB94, or combination for 2 h upon 48 h Mtb infection by Western blot analysis and CFU assay (means ± SD, *n* = 3 independent experiments with each 4 replicates). Mtb-infected mice were treated with DMSO (*n* = 4), SB216763 (20 mg/kg, *n* = 4), SB3CT (20 mg/kg, *n* = 4), and combination of SB216763 with SB3CT (20 mg/kg, *n* = 4) for 4 weeks. **(K)** MMP-9 expression in the supernatant of lungs and spleens were detected by ELISA analysis. **(L)** H37Rv infection of lungs and spleens were detected by CFU assay (means ± SD, *n* = 3 independent experiments with each 4 replicates). For Western blot assay, GAPDH served as an internal control. Data presented are from one of at least three independent experiments with similar results. The numbers below immunoblot correspond to band-integrated density ration of target protein to GAPDH. *p ≤ 0.05 and **p ≤ 0.01 were considered statistically significant. BB94, MMP inhibitor; SB3CT, MMP-9 inhibitor; SB216763, GSK-3α/β inhibitor.

Next, we found that Mtb infection significantly increased intracellular and supernatant MMP-1 and MMP-9 expressions in a time-dependent manner in THP-1-Mφs (**Figure 3A**). Increased MMP-9 protein expression has also been observed in lungs of six patients with pulmonary tuberculosis and in lymph nodes of six patients with lymphatic tuberculosis, compared with that in patients of chronic inflammation by immunohistochemistry analysis (**Figure 3B**). These results suggested that MMP-1/9 might play key roles in host response against Mtb infection. We applied BB94, which is a broad spectrum for MMPs including MMP-1/9, to further explore direct effect of MMP-1/9 on tuberculosis. As expected, BB94 reduced Mtb-induced intracellular and supernatant MMP-1 and MMP-9 expressions (**Figure 3C**). CFU assay showed that BB94 treatment significantly decreased intracellular Mtb infection (**Figure 3D**). We exclude the possibility that BB94 inhibited cell growth to suppress Mtb infection because BB94 treatment did not affect cell proliferation detected by CCK-8 assay (**Supplementary Figure S1A**). BB94 did not decrease Mtb infection directly as detected by CFU assay (**Supplementary Figure S1B**). Three siRNAs targeting MMP-1/9 were transfected in THP-1-Mφs, and MMP-1/9 silencing showed potent decrease of MMP-1/9 mRNA and protein expression (**Figure 3E**). CFU assays showed that MMP-1/9 silencing significantly decreased bacterial load in THP-1-Mφs at 48 h postinfection (**Figure 3F**). Correspondingly, overexpression of MMP-1, MMP-2, and MMP-9 in THP-1-Mφs showed a significant increase of MMP-1, MMP-2, and MMP-9 expression (**Figure 3G**), but only MMP-1 and MMP-9 overexpression showed a significant increase of Mtb infection detected by CFU assay (**Figure 3H**). Next, the association of GSK-3α/β with MMP-1/9 in Mtb-infected Mφs and the effect of MMP-1/9 on Mtb infection prompted us to further explore whether GSK-3α/β inhibits Mtb infection through inhibiting MMP-1/9 expression. Our results showed that BB94 obviously diminished MMP-1/9 protein expression promoted by SB216763 treatment (**Figure 3I**). Similarly, BB94 treatment significantly blocked the increased intracellular Mtb infection by SB216763 treatment in THP-1-Mφs upon 48 h of Mtb infection by CFU assay (**Figure 3J**). These results suggest that GSK-3α/β activity negatively regulates MMP-1/9 production to facilitate Mtb infection in Mφs.

As mice do not express MMP-1 orthologue, we further investigated whether the effect of GSK-3α/β on Mtb infection is related to MMP-9 expression in mice (9). Treating with GSK-3α/β inhibitor SB216763 for 4 weeks significantly increased MMP-9 production in the supernatant of lungs and spleens of Mtb-infected mice, whereas MMP-9 inhibitor SB3CT-blocked SB216763 increased MMP-9 expression (**Figure 3K**). Consistently, CFU assay showed that SB3CT significantly decreased bacterial load in the lungs and spleens after 4 weeks of Mtb infection and diminished Mtb infection increased by SB216763 treatment (**Figure 3L**). However, SB3CT did not directly affect Mtb infection as detected by CFU assay (**Supplementary Figure S1B**). These results indicated that GSK-3α/β suppresses Mtb infection through inhibiting MMP-9 expression in mice.

### GSK-3α/β Suppresses MMP-1/9 Expression to Inhibit Mtb Infection Through Phospho-ERK1/2

**Figure 4:**
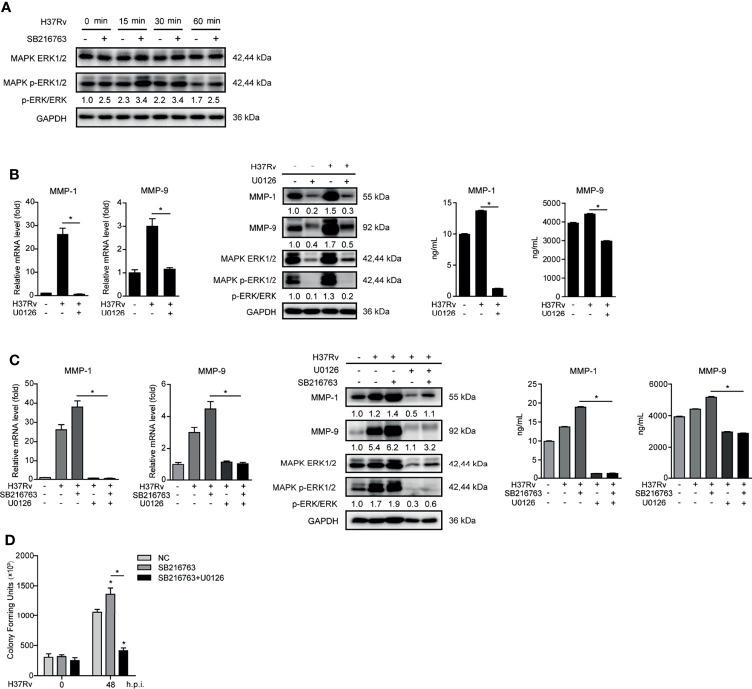
GSK-3α/β suppressed MMP-1/9 expression to inhibit Mtb infection through phospho-ERK1/2. **(A)** Phosphorylation of GSK-3α/β (Ser21/9 and Tyr216/279) and ratio of total GS to phosphorylated GS were detected 2 h pretreated with U0126 in THP-1-Mφs upon Mtb infection at 48 h by Western blot analysis. Intracellular mRNA, protein expression, and secretion of MMP-1/9 were analyzed in THP-1-Mφs **(B)** pretreated with 10 μM U0126 or **(C)** pretreated with 20 μM SB216763, 10 μM U0126, and combination of both for 2 h at 48 h.p.i. by qRT-PCR, Western blot, and ELISA analysis. Expression of ERK1/2 and phospho-ERK1/2 were detected by Western blot analysis (means ± SD, *n* = 3 independent experiments with each 4 replicates). **(D)** Intracellular Mtb load was measured by CFU assays at 48 h.p.i. (means ± SD, *n* = 3 independent experiments with each 4 replicates). For Western blot assay, GAPDH served as an internal control. Data presented are from one of at least three independent experiments with similar results. The numbers below immunoblot correspond to band-integrated density ration of target protein to GAPDH. ^*^ *p* ≤ 0.05 was considered statistically significant. U0126, ERK1/2 inhibitor; SB216763, GSK-3α/β inhibitor.

MAPK ERK1/2 is a key regulatory factor in controlling MMP-1/9 expression (21). We found that SB216763 treatment markedly enhanced phosphorylation of ERK1/2 upon Mtb infection at different time points in THP-1-Mφs (**Figure 4A**). To investigate whether GSK-3α/β suppresses MMP-1/9 expression and Mtb infection through ERK1/2 phosphorylation, we used ERK1/2 inhibitor U0126 to treat THP-1-Mφs. The results showed that inhibition of ERK1/2 phosphorylation dramatically suppressed intracellular and supernatant MMP-1/9 expression upon Mtb infection for 48 h (**Figure 4B**). Most importantly, intracellular and supernatant MMP-1/9 expressions induced by SB216763 were totally abolished by U0126 treatment (**Figure 4C**). Consistently, U0126 treatment blocked the increase of Mtb infection mediated by SB216763 treatment at 48 h.p.i. (**Figure 4D**). U0126 or its combination with SB216763 did not affect cell proliferation determined by CCK-8 assay (**Supplementary Figure S1A**). U0126 and SB216763 did not directly influence Mtb infection as detected by CFU assay (**Supplementary Figure S1B**). These results suggest that GSK-3α/β exerts antimicrobial effect through negatively regulating ERK1/2 phosphorylation and subsequent MMP-1/9 expression in Mφs.

### MTOR Inhibits GSK-3α/β Activity to Promote Phospho-ERK1/2 Mediated MMP-1/9 Expression During Infection

**Figure 5:**
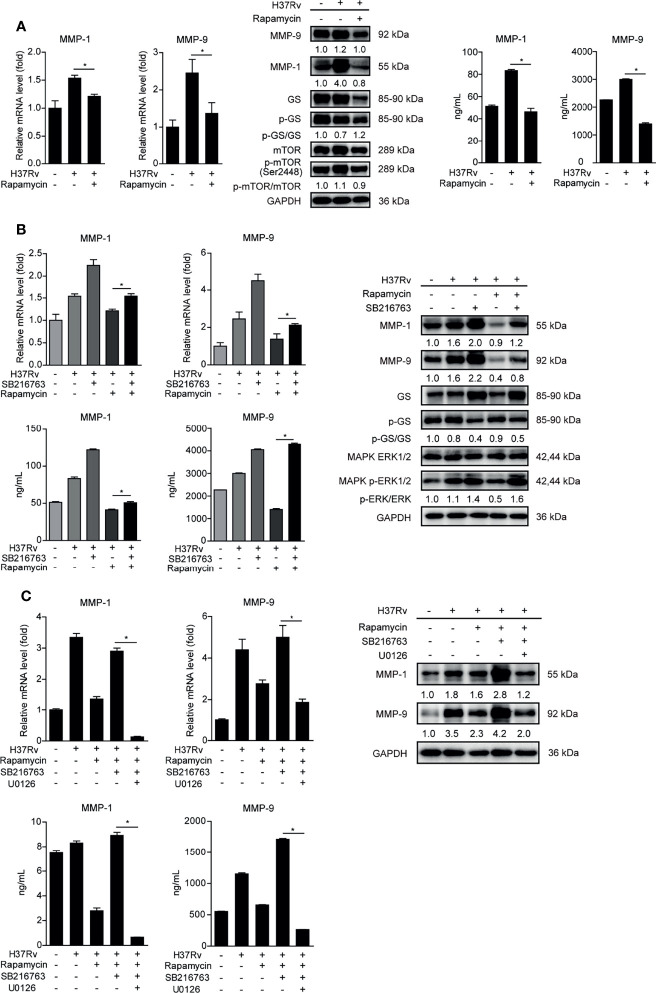
MTOR inhibits GSK-3α/β activity to promote phospho-ERK1/2 mediated MMP-1/9 expression during infection. Intracellular mRNA, protein expression, and secretion of MMP-1/9 were analyzed in THP-1-Mφs with 2 h pretreatment with **(A)** 1 μM rapamycin, **(B)** SB216763, rapamycin, or their combination, **(C)** SB216763, U0126, rapamycin, or their combination by qRT-PCR, Western blot, and ELISA analysis at 48 h postinfection. Expression of GS, ERK1/2, mTOR, and phosphorylation of GS, ERK1/2, and mTOR were detected by Western blot analysis (means ± SD, *n* = 3 independent experiments with each 4 replicates). For Western blot assay, GAPDH served as an internal control. Data presented are from one of at least three independent experiments with similar results. The numbers below immunoblot correspond to band integrated density ration of target protein to GAPDH. ^*^ *p* ≤ 0.05 was considered statistically significant. U0126, ERK1/2 inhibitor; rapamycin, mTOR inhibitor; SB216763, GSK-3α/β inhibitor.

MTOR has been reported as an important moderating factor of GSK-3α/β activity (22). To investigate whether mTOR participates in regulating GSK-3α/β activity and MMP-1/9 expression, we pretreated THP-1-Mφs with rapamycin of mTOR inhibitor. The results showed that rapamycin suppressed mTOR phosphorylation and intracellular and supernatant MMP-1/9 expressions but promoted GSK-3α/β activity indicated by the increased ratio of activated GS to total GS (**Figure 5A**). Furthermore, we demonstrated that MMP-1/9 expression and ERK1/2 phosphorylation inhibited by rapamycin were restored with SB216763 treatment while rapamycin increased GSK-3α/β activity was reduced with SB216763 treatment (**Figure 5B**). Moreover, the recovery effect of SB216763 treatment on MMP-1/9 expression inhibited by rapamycin was further blocked by U0126 treatment (**Figure 5C**). These results were found not to be related to regulatory cell proliferation with SB216763, rapamycin, or/and U0126 treatment (**Supplementary Figure S1A**). These results suggested that mTOR negatively regulated GSK-3α/β activity to promote ERK1/2 phosphorylation mediated MMP-1/9 expression in Mtb-infected Mφs.

### PKC-δ-mTORC Axis Negatively Regulated GSK-3α/β Activity to Promote Phospho-ERK1/2 Mediated MMP-1/9 Expression

**Figure 6:**
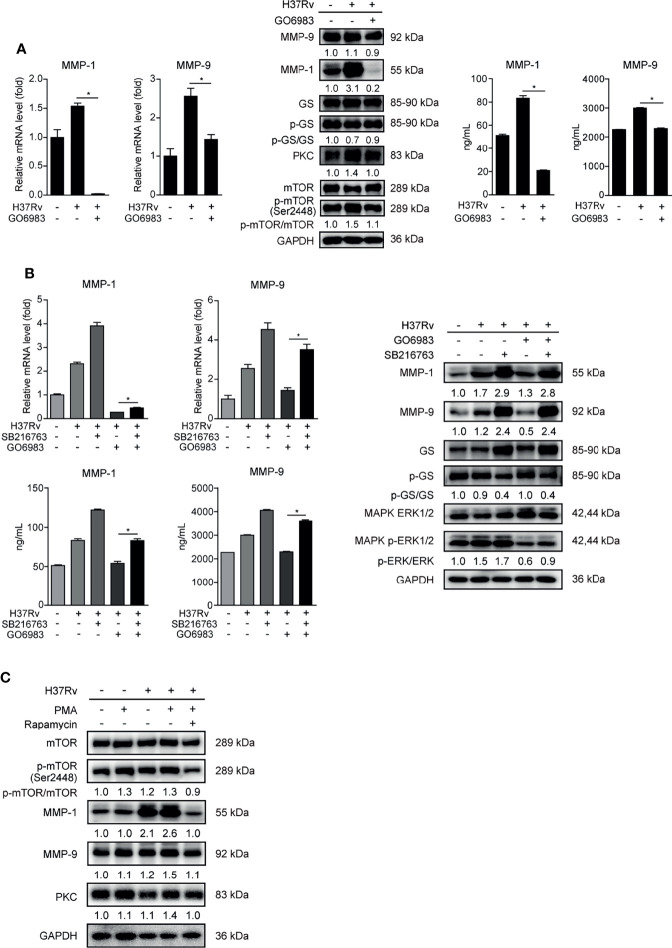
PKCs play a key role in mTOR-regulated GSK-3α/β activity in THP-1-Mφs. Intracellular mRNA, protein expression, and secretion of MMP-1/9 were analyzed in THP-1-Mφs with 2 h pretreatment with **(A)** 1 μM GO6983 and **(B)** SB216763, GO6983, or their combination at 48 h postinfection by qRT-PCR, Western blot, and ELISA analysis. Protein expression of GS, ERK1/2, mTOR, and PKC and phosphorylation of GS, ERK1/2, and mTOR were detected by Western blot analysis (means ± SD, *n* = 3 independent experiments with each 4 replicates). **(C)** Protein expression of MMP-1/9, mTOR, and PKC and phosphorylation of mTOR were detected in THP-1-Mφs pretreated with PMA or the combination of PMA with rapamycin at 48 h.p.i. by Western blot analysis. For Western blot assay, GAPDH served as an internal control. Data presented are from one of at least three independent experiments with similar results. The numbers below immunoblot correspond to band integrated density ration of target protein to GAPDH. ^*^ *p* ≤ 0.05 was considered statistically significant. GO6983, PKC inhibitor; PMA, PKC activator; SB216763, GSK-3α/β inhibitor.

It has recently been reported that PKC kinase activity is required for mTOR activation (23), thus we further studied the relationship between PKC and mTOR in regulating GSK-3α/β activity and MMP-1/9 expression. We exploited pan-PKC inhibitor GO6983 to treat THP-1-Mφs. We observed that GO6983 increased the ratio of activated GS to total GS, and it reduced PKC expression, phosphorylation of mTOR and MMP-1/9 expression, indicating that PKCs function upstream of mTOR signaling to regulate GSK-3α/β activity and MMP-1/9 expression (**Figure 6A**). SB216763-abrogated GO6983 enhanced GSK-3α/β activity and restored GO6983-suppressed ERK1/2 phosphorylation and MMP-1/9 expression (**Figure 6B**). Consistently, PKC agonist PMA increased mTOR phosphorylation and MMP-1/9 expression; however, such elevation of MMP-1/9 expression was blocked by rapamycin treatment (**Figure 6C**). These results were also not related to cell proliferation (**Supplementary Figure S1A**).

**Figure 7:**
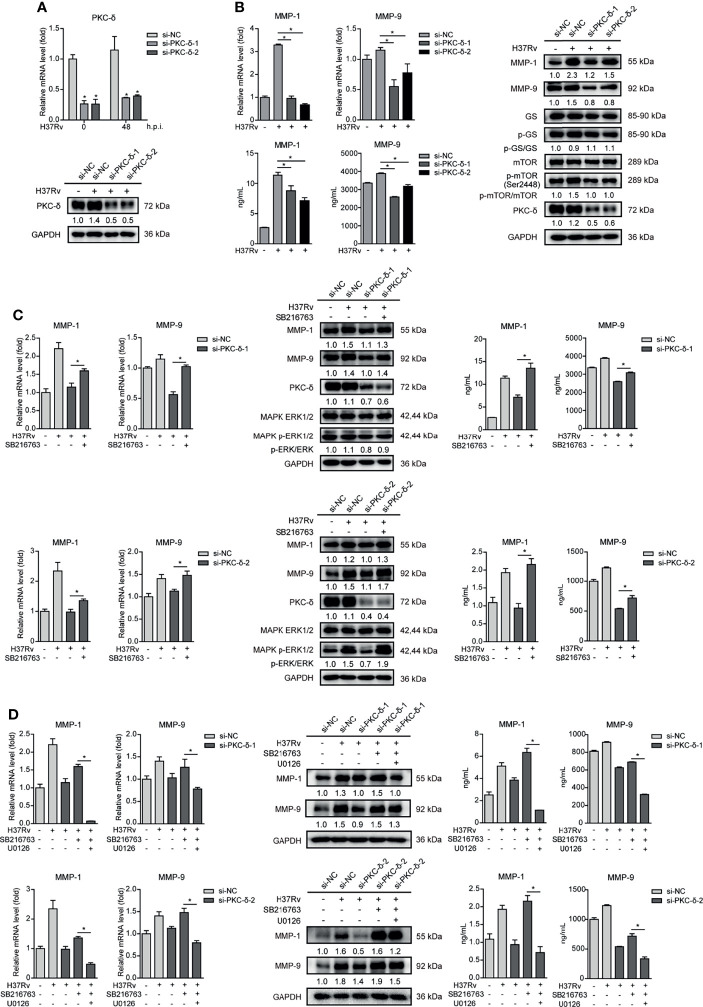
PKC-δ-mTORC axis negatively regulated GSK-3α/β activity to promote phospho-ERK1/2 mediated MMP-1/9 expression. **(A)** PKC-δ expression of mRNA and protein levels were determined in PKC-δ silenced THP-1-Mφs upon 48 h Mtb infection by qRT-PCR and Western blot analysis (means ± SD, *n* = 3 independent experiments with each 4 replicates). Intracellular mRNA, protein expression, and secretion of MMP-1/9 were analyzed in **(B)** silencing PKC-δ, **(C)** silencing PKC-δ, SB216763, or their combination, **(D)** silencing PKC-δ, SB216763, U0126, or their combination in THP-1-Mφs at 48 h postinfection by qRT-PCR, Western blot, and ELISA analysis. Protein expression of GS, mTOR, ERK1/2, and PKC-δ and phosphorylation of GS, mTOR, and ERK1/2 were detected by Western blot analysis (means ± SD, *n* = 3 independent experiments with each 4 replicates). For Western blot assay, GAPDH served as an internal control. Data presented are from one of at least three independent experiments with similar results. The numbers below immunoblot correspond to band-integrated density ration of target protein to GAPDH. ^*^ *p* ≤ 0.05 was considered statistically significant. U0126, ERK1/2 inhibitor; SB216763, GSK-3α/β inhibitor.

Such results prompted us to further explore which PKC subtypes affect GSK-3α/β activity and MMP-1/9 expression. We used different siRNAs targeting various PKC subtypes to knock down PKC-α, PKC-β, PKC-γ, PKC-δ, and PKC-ϵ, respectively, and detected mRNA expression of them in THP-1-Mφs upon Mtb infection. All of them were silenced by their own siRNAs according to significant decrease of mRNAs of PKC-α, PKC-β, PKC-γ, PKC-δ, and PKC-ϵ (**Supplementary Figure S4** and **Figure 7A**). Among these PKC subtypes, only silencing of PKC-δ apparently suppressed mRNA expression of MMP-1/9, while silencing of other PKC subtypes showed no or little effect on MMP-1/9 expression (**Supplemental Figure S4**). We further confirmed that PKC-δ silencing decreased intracellular and supernatant MMP-1/9 expression and mTOR phosphorylation and increased GSK-3α/β activity (**Figures 7A, B**). Furthermore, we found that SB216763 treatment restored ERK1/2 phosphorylation and reduced MMP-1/9 expression *via* PKC-δ silencing (**Figure 7C**). Importantly, such recovery effects of SB216763 on MMP-1/9 expression was further blocked by U0126 treatment (**Figure 7D**). Thus, we conclude that PKC-δ-mTOR negatively regulated GSK-3α/β activity to promote phospho-ERK1/2 mediated MMP-1/9 expression during Mtb infection in Mφs.

## Discussion

**Figure 8:**
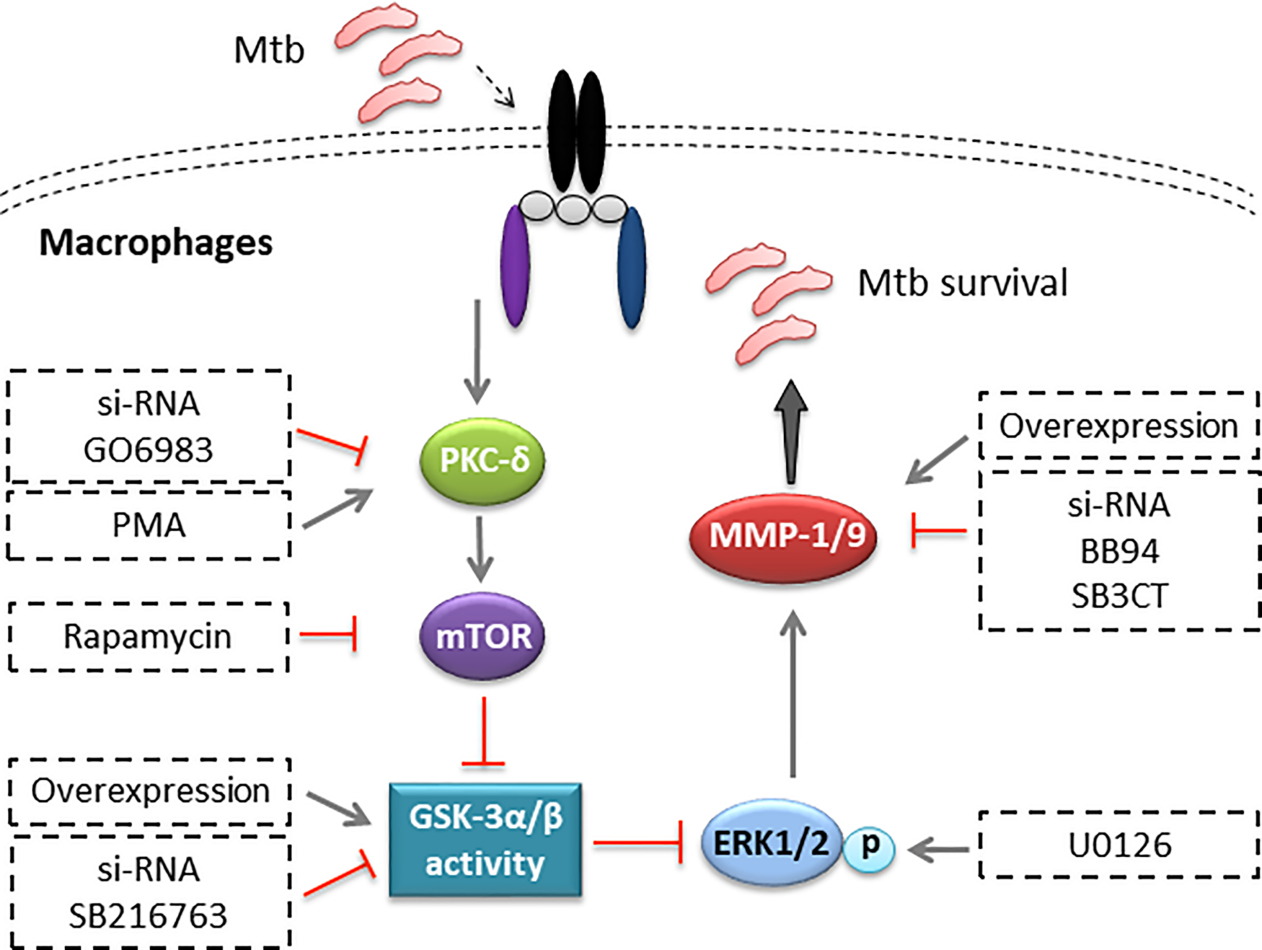
Illustration of model of PKC-δ-mTOR axis negatively regulated GSK-3α/β activity, inhibiting Mtb infection through phospho-ERK1/2 mediated MMP-1/9 expression in Mφs.

While Mφs and neutrophils represent the first-line responders to Mtb infection, they also provide a major habitat for Mtb to reside in the host. Through a long-term battle with the host, Mtb develops various strategies to regulate host factors and counter the bactericidal activity of the host’s immunity, resulting in its survival and proliferation in Mφs (24). In the present study, we find that Mtb infection downregulates GSK-3α/β activity and upregulates MMP-1/9 expression in THP-1-Mφs. MMP-9 expression remarkably increases in both lungs of patients with pulmonary tuberculosis and lymph nodes of patients with lymphatic tuberculosis. However, it cannot be excluded that upregulated MMP-9 was secreted by other host immune cells such as neutrophils in both lung and lymph nodes of TB patients. Macrophage marker can be costained with MMP-9 protein to identify that macrophage-secreted MMP-9 by TB patients for further study. These results are in agreement with previous observations that Mtb tyrosine phosphatase PtpA dephosphorylated GSK-3α to modulate host Mφs immune responses against Mtb infection (7). Our results also support the findings that MMP-9 expression was strongly upregulated in TB lesions and distal regions of the lung biopsies (25). Additionally, MMP-1 was demonstrated as the principal secreted collagenase and upregulated in the sputum and bronchoalveolar lavage fluid of TB patients (9). Further investigation identify that Mtb infection promotes MMP-1/9 expression, which further facilitates Mtb infection, while GSK-3α/β suppresses Mtb infection through inhibiting MMP-1/9 expression in Mφs and mice (**Figure 8**). Taken together, these findings have provided a new perspective as how Mtb escapes from host immune responses. However, it is still unknown whether heat-killed Mtb or avirulent mutants including an ESX-1 mutant and an ESX-5 mutant can evade the immune responses as an active strategy. In addition, how Mtb initiates the signaling pathway in Mφs remains unclear in this study. Specific ligands including TLR2, TLR4, TLR9, and cGAS-STING agonists should be applied for further investigation.

As a multifunctional kinase involved in an array of critical cellular processes, GSK-3α/β deserved an important component of host defense system (3). Recent studies, for example, have shown that GSK-3β promotes Mφs inflammatory activation by inhibiting the immune regulatory signaling of AMP-activated protein kinase (AMPK) (26). Human natural killer (NK) cells significantly increased production of TNF and IFN-γ, elevated natural cytotoxicity, and increased antibody-dependent cellular cytotoxicity in the presence of a GSK-3α/β inhibitor *ex vivo* (27). GSK-3β could interact with PD-L1 and play an important role in antitumor T-cell immunity of breast cancer (28). Importantly, autophagy machinery and inflammatory response, representative pivotal host immune strategies in combating Mtb infection, have been identified under the regulation of GSK-3α/β (3, 29–32). In our study, we have detected autophagy and proinflammatory and anti-inflammatory cytokines including IFNs and ISGs upon Mtb infection with treatment of SB216763 in THP-1-Mφs. Autophagy was not regulated by SB216763 treatment in a time-dependent manner. We know that IL-6, TNF-α, and IL-1β are proinflammatory cytokines which can play anti-Mtb infection role and IL-10 is an anti-inflammatory cytokine which can promote Mtb infection. Here, we demonstrated that expression of IL-6 and TNF-α significantly decreased 48 h.p.i.; however, expression of IL-1β and IL-10 significantly increased 24 and 48 h.p.i. with SB216763 treatment. Therefore, whether GSK-3α/β regulates Mtb infection though these cytokines needs to be further investigated. Expression of IFN-α and IFN-γ, as well as Mx1, Rsad2, and ISG15, was not influenced by SB216763 treatment. IFN-β and IRF1 expression was significantly decreased at 48 h.p.i. IFN-β production plays a probacterial role in host-Mtb interactions (33). Our previous study showed that IRF1 did not affect MMP expression upon Mtb infection (20). Thus, we speculate that autophagy, inflammatory cytokine production, and IFN signaling are not the main regulators of GSK-3α/β to suppress Mtb infection in Mφs.

Several MMPs stimulated by Mtb infection have been associated with the initiation and progression of TB. Among them, MMP-1 and MMP-9 are most extensively studied owing to their roles in the creation of the granuloma and destruction of lung tissue (10, 34). It has been reported that MMP-1 plays an important role in the immunopathology of TB. It promotes the collagen breakdown that lead to pulmonary tissue destruction in TB (9). Other studies have shown that MMP-9 enhanced the recruitment of newer Mφs and was associated with nascent granuloma maturation and bacterial growth (35). Previous studies showed that mice treated with MMP inhibitor BB94 exhibited either a delay in granuloma induction (36, 37) or formed smaller granulomas with more collagen (38), suggesting the crucial role of MMPs in regulating cell migration and granuloma formation upon Mtb infection. Furthermore, adjunctive treatment with MMP inhibitors along with front-line anti-TB drugs including isoniazid and rifampin significantly reduced Mtb survival in the lungs by preventing maturation of granulomas and minimizing the matrix degradation and cavitary lesions (39–41). This new regimen of improved TB treatment by inhibition of MMP activity will help with minimizing the TB-associated morbidity and mortality. Consistently, our current study demonstrates that Mtb infection promoted MMP-1/9 expression and significantly facilitates Mtb survival in Mφs. MMP-9 inhibitor of SB3CT exhibits a significant decrease of Mtb infection in lungs and spleens of mice. Although the potential specific mechanism of MMPs on Mtb infection needs further studies, our study makes a crucial sense in revealing the importance of MMP-1/9 in regulating the immune pathology of Mtb infection.

MMPs have been demonstrated as can be regulated by different signaling pathways especially GSK-3α/β, ERK1/2, JNK, p38 MAPK, or/and NF-κB/AP-1 activation in various diseases (15, 16, 42–44). GSK-3β-regulated MMP expression, for example, was involved in SLFN5-controlled inhibition of cancer cell migration and invasion (45). MMP-9 expression mediated by dysregulation of GSK-3β activity has dramatic consequences on synaptic alterations and dendritic spine morphology (46). In addition, TNF-α and GM-CSF-induced GSK-3α/β inhibition determined the increase of MMP-1 production through a mechanism involving ERK1/2 activation by monocytes (47). Similarly, GSK-3β inhibition mediated ERK1/2 activation followed by the induction of MMP-9 expression in rat primary astrocytes (13). In our study, we find a novel mechanism for GSK-3α/β activity mediated ERK1/2 phosphorylation to exert anti-Mtb effect through suppressing MMP-1/9 expression in Mφs.

Given the fact that GSK-3α/β lies in the crossroads of various signal pathways, we have explored the upstream regulators of GSK-3α/β in Mtb infection. PKC and mTOR pathways represent the most common pathways implicated in the regulation of GSK-3α/β activity. For example, PKC suppressed GSK-3α/β activity, resulting in ERK1/2 phosphorylation, which was essential for MMP-1 production from monocytes (47). Inhibition of mTOR attenuated GSK-3β activity to increase NF-κB p65-associated CREB-binding protein and modulate balance of pro- and anti-inflammatory cytokines (17). In agreement with these findings, this study demonstrated that PKC-δ-mTOR axis promotes ERK phosphorylation-mediated MMP-1/9 expression through suppressing GSK-3α/β activity in Mφs. However, some studies have shown results completely opposite to those of our study regarding how mTOR activity affects MMP expression during Mtb infection (48). These differences might be due to the type of Mφs studied. The exact mechanisms associated with these differences require further investigation. Interestingly, it was reported that GSK-3α/β is involved in the regulation of PKC-δ and mTOR activity. GSK-3α/β decreased PKC-δ activity, attenuating the induction of ERK1/2 phosphorylation by GSK-3α/β inhibition (12). Moreover, GSK-3β regulates mTOR activity as well as in cancer research in hepatocellular carcinoma (HCC) (49). Our study mainly indicated that the PKC-δ-mTOR axis inhibits the activity of GSK-3α/β through ERK1/2 phosphorylation to upregulate the expression of MMP-1/9; the in-depth mechanism whether GSK-3α/β has a negative feedback mechanism for PKC-δ and mTOR requires further research.

In conclusion, we demonstrate that Mtb can escape from host immunity by suppressing GSK-3α/β activation and promoting MMP-1/9 production. Furthermore, GSK-3α/β activity regulated by PKC-δ-mTOR axis inhibits Mtb infection through suppressing phospho-ERK1/2-mediated MMP-1/9 expression in Mφs. This study sheds new light on the molecular insight of host-Mtb interaction and bears significant implications in the development of novel therapeutic approaches for TB.

## Data Availability Statement

The raw data supporting the conclusions of this article will be made available by the authors, without undue reservation.

## Ethics Statement

The studies involving human participants were reviewed and approved by the Ethics Committee of the Southern Medical University. The patients/participants provided their written informed consent to participate in this study. The animal study was reviewed and approved by the Ethics Committee of Southern Medical University.

## Author Contributions

Conceptualization: XZ and LM. Methodology: XZ and LL. Software: XZ and LL. Validation: XZ, LL, and LM. Formal analysis: XZ and LL. Investigation: XZ, LL, YL, HX, BZ, YiH, LZ, ZZ, QL, QWa, ZH, YuH, HL, SH, CZ, and QWe. Resources: XZ, LL, SH, CZ, QWe, and LM. Data curation: XZ and LL. Writing—original draft preparation: XZ, LL, and L. Writing—review and editing: XZ, LL, and LM. Visualization: XZ and LL. Supervision: XZ and LM. Project administration: XZ and LM. Funding acquisition: XZ and LM. All authors read and approved the final manuscript.

## Funding

This research was funded by the National Natural Science Foundation of China (81772150, 82072242, 82070010, 81801584, 81800013) and Guangdong Basic and Applied Basic Research Foundation (2021A1515010933, 2019A1515010988, 2018030310486).

## Conflict of Interest

The authors declare that the research was conducted in the absence of any commercial or financial relationships that could be construed as a potential conflict of interest.

## Publisher’s Note

All claims expressed in this article are solely those of the authors and do not necessarily represent those of their affiliated organizations, or those of the publisher, the editors and the reviewers. Any product that may be evaluated in this article, or claim that may be made by its manufacturer, is not guaranteed or endorsed by the publisher.
