# Supplementary material for: GSK-3α/β Activity Negatively Regulates MMP-1/9 Expression to Suppress Mycobacterium tuberculosis Infection
Source: Front Immunol. 2022 Jan 12;12:752466. doi: 10.3389/fimmu.2021.752466 (PMC8789754; doi:10.3389/fimmu.2021.752466)
Supplement: Supplementary file 1 [file DataSheet_1.docx]

GSK-3α/β activity negatively regulates MMP-1/9 expression to suppress *Mycobacterium tuberculosis* infection

SUPPLEMENTARY MATERIALS

Supplemental Figures 1-4

Supplemental Table 1


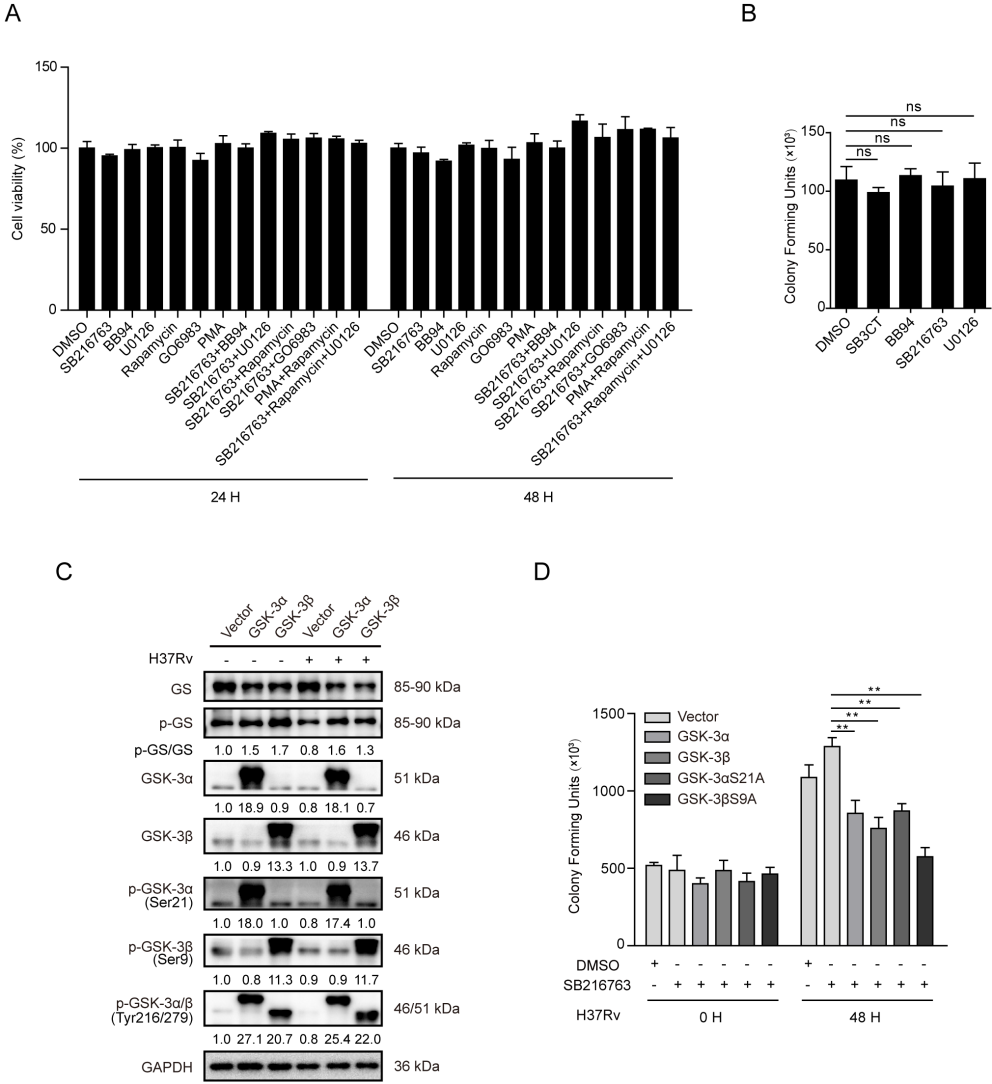


**Supplemental Figure 1.** Treatment of different reagents or their combinations have no cytotoxicity on THP-1-Mφs and overexpression of GSK-3α, GSK-3αS21A, GSK-3β and GSK-3βS9A suppresses SB216763 promoted Mtb infection. **(A)** THP-1-Mφs were 2 h pretreated with SB216763, BB94, U0126, rapamycin, GO6983, PMA, or their combinations, cytotoxicity was detected by CCK-8 assay at 24 h and 48 h. Treatment of DMSO at the same concentration was normalized to 100% (means ± SEM, n = 3 independent experiments with each 3 replicates). **(B)** The direct effect of inhibitors including SB216763, BB94, SB3CT and U0126 on Mtb infection was detected by CFU assay at 48 h.p.i. (means ± SD, n = 3 independent experiments with each 4 replicates). **(C)** Protein expression of GSK-3α/β, phospho-GSK-3α/β (Ser21/9), phospho -GSK-3α/β (Tyr216/279), GS and phospho-GS was detected in GSK-3α/β overexpressed THP-1-Mφs upon Mtb infection at 48 h by Western blot analysis. GAPDH served as an internal control. Data presented are from one of at least three independent experiments with similar results. **(D)** Intracellular H37Rv load was measured in THP-1-Mφs overexpressing GSK-3α, GSK-3αS21A, GSK-3β or GSK-3βS9A with 2 h pretreatment of SB216763 upon Mtb infection at 48 h by CFU assay (means ± SD, n = 3 independent experiments with each 4 replicates). *p ≤ 0.05 and **p ≤ 0.01. Ns, not significant. SB216763: GSK-3α/β inhibitor; BB94, MMPs inhibitor; U0126: ERK1/2 inhibitor; Rapamycin: mTOR inhibitor; GO6983: PKC inhibitor; PMA: PKC activator; SB3CT: MMP-9 inhibitor.


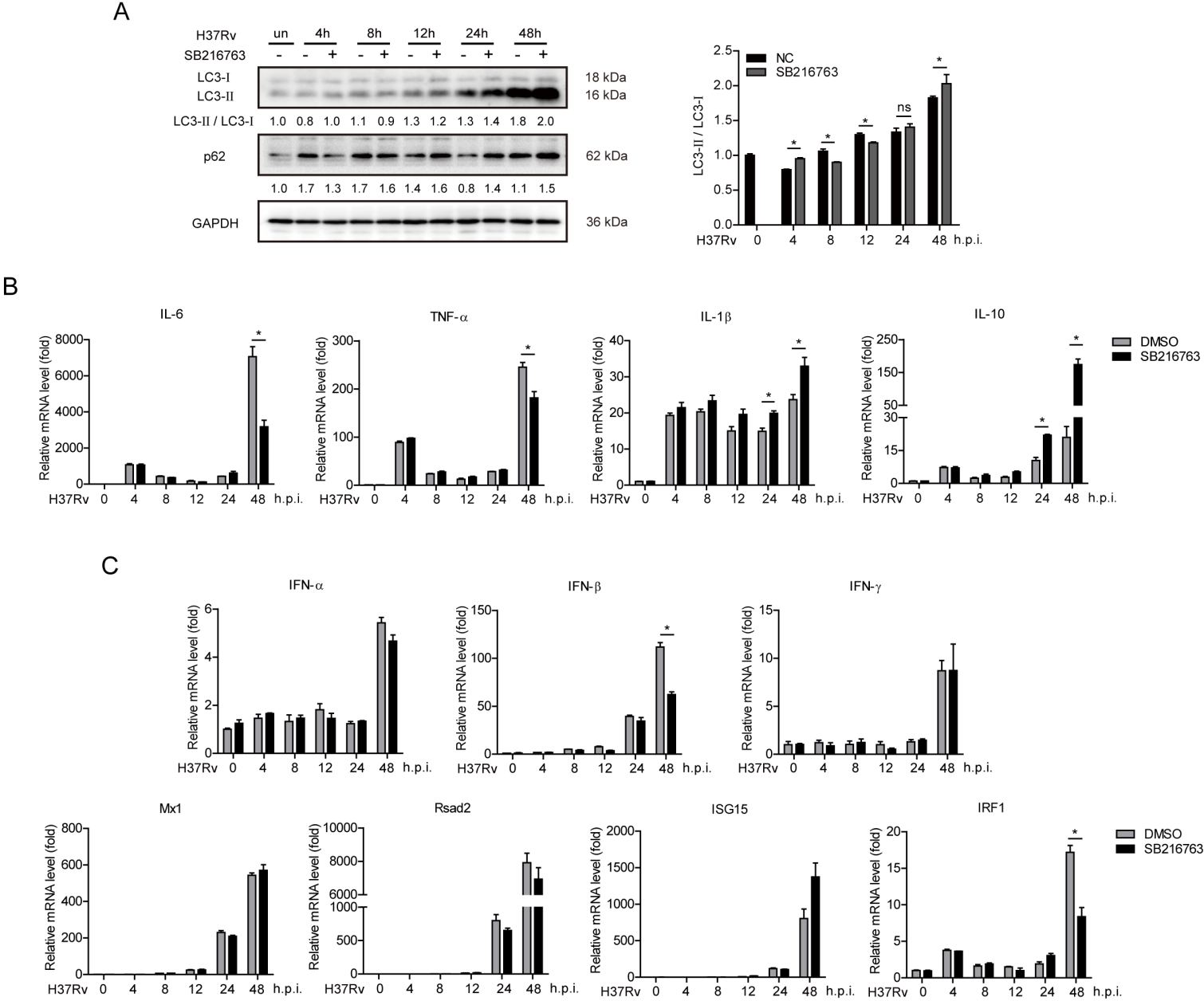


**Supplemental Figure 2.** Autophagy, inflammatory cytokines and ISGs are irregularly influenced by SB216763 treatment during Mtb infection. **(A)** Protein expression of LC3-I and -II was detected in THP-1-Mφs pretreated with SB216763 for 2 h following 4, 8, 12, 24 and 48 h Mtb infection by Western blot analysis. The conversion of LC3-I to LC3-II was indicated by ratio of LC3-II/ LC3-I expression. GAPDH served as an internal control. Data presented are from one of at least three independent experiments with similar results. **(B)** mRNA expression of inflammatory factors of IL-6, TNF-α, IL-1β and IL-10 and **(C)** mRNA expression of IFN-α, -β, -γ, Mx1, Rsad2, ISG15 and IRF1 was determined in THP-1-Mφs pretreated with SB216763 for 2 h following Mtb infection at indicated time points by qRT-PCR. GAPDH served as reference gene (means ± SD, n = 3 independent experiments with each 4 replicates). *p ≤ 0.05. SB216763: GSK-3α/β inhibitor.

**
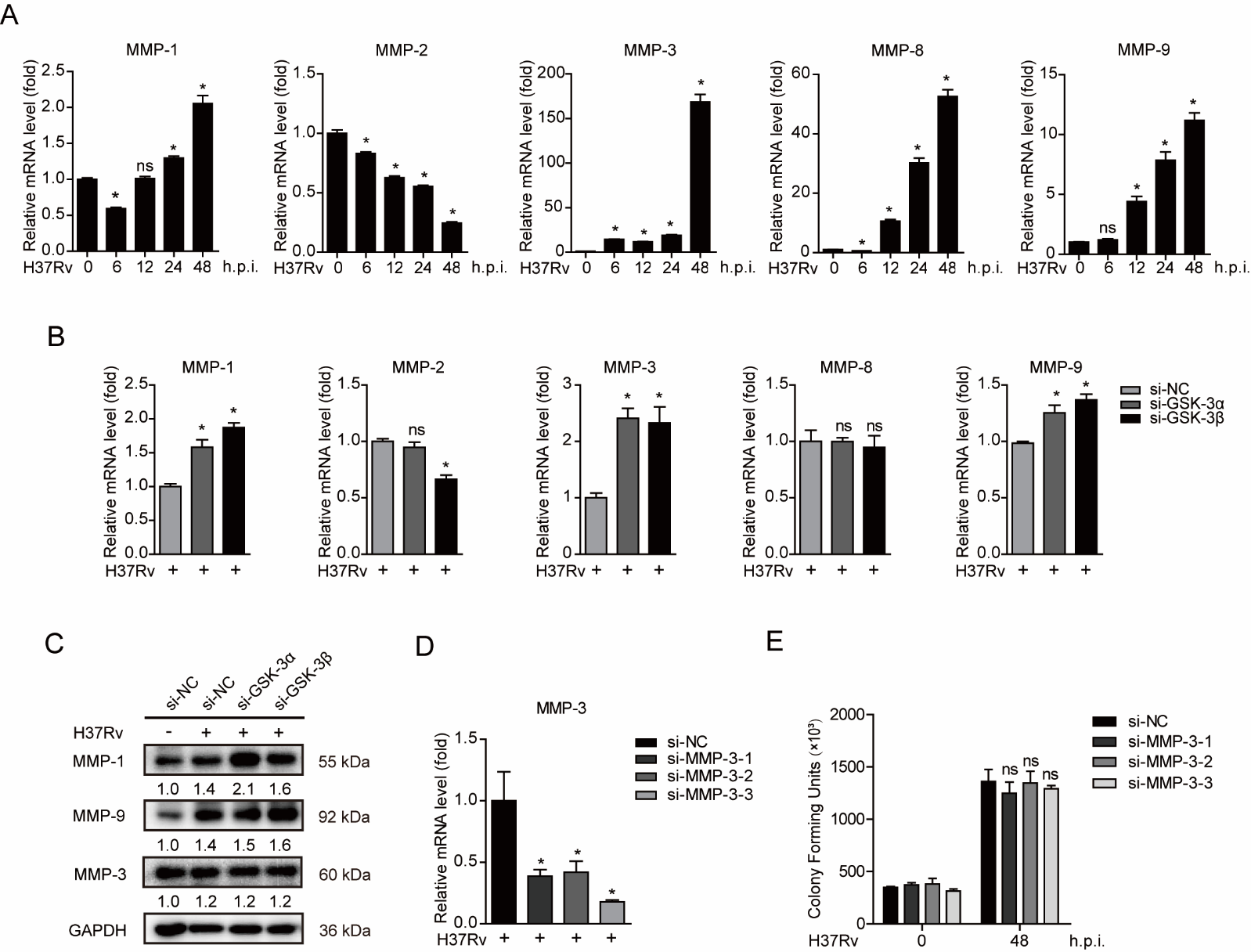
Supplemental Figure 3.** The expression of MMP-1, -2, -3, -8 and -9 in THP-1-Mφs with Mtb infection or in GSK-3α and -3β silenced THP-1-Mφs, and the effect of MMP-3 silencing on Mtb infection. **(A)** mRNA expression of MMP-1, -2, -3, -8 and -9 was determined in THP-1-Mφs upon Mtb infection at indicated time points by qRT-PCR assay. GAPDH served as reference gene (means ± SD, n = 3 independent experiments with each 3 replicates). **(B)** GSK-3α and -3β silenced THP-1-Mφs were detected for MMP-1, -2, -3, -8 and -9 mRNA expression by qRT-PCR upon 48 h of Mtb infection (means ± SD, n = 3 independent experiments with each 3 replicates). **(C)** MMP-1, -3 and -9 expression was detected in GSK-3α and -3β silenced THP-1-Mφs upon Mtb infection at 48 h by Western blot analysis. GAPDH served as an internal control. Data presented are from one of at least three independent experiments with similar results. **(D)** MMP-3 mRNA expression was detected in MMP-3 silenced THP-1-Mφs at 48 h.p.i. by qRT-PCR assay (means ± SD, n = 3 independent experiments with each 2 replicates). **(E)** CFU assay was applied to detect intracellular Mtb load in MMP-3 silenced THP-1-Mφs at 48 h.p.i. (means ± SD, n = 3 independent experiments with each 4 replicates). *p ≤ 0.05. Ns, not significant.


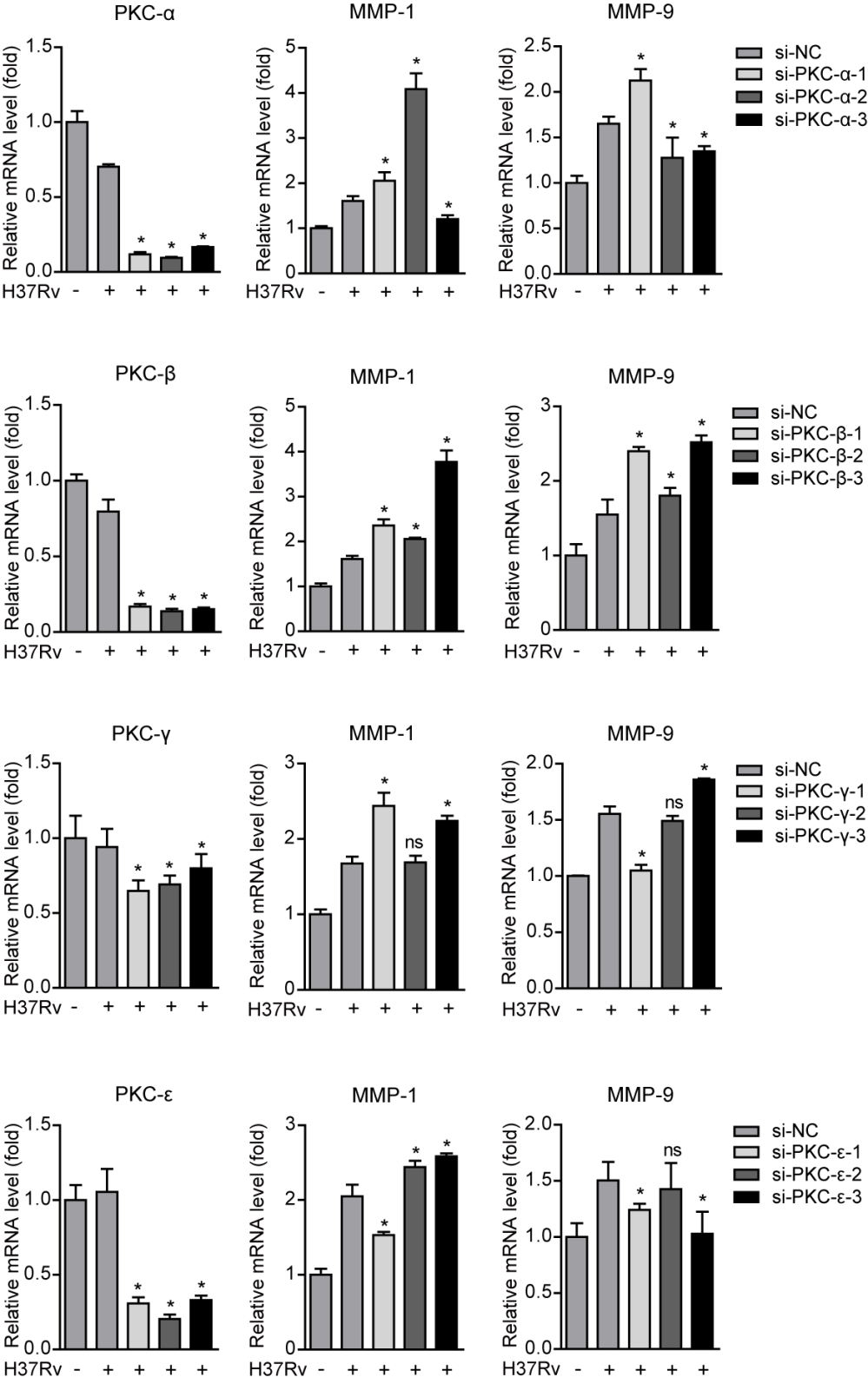


**Supplemental Figure 4.** MMP-1/9 expression was detected in THP-1-Mφs with PKC-α, PKC-β, PKC-γ and PKC-ε silencing. THP-1-Mφs transfected with three si-RNAs targeting PKC-α, PKC-β, PKC-γ and PKC-ε with Mtb infection for 48 h. PKC-α, PKC-β, PKC-γ, PKC-ε and MMP-1/9 mRNA expression was detected by qRT-PCR. GAPDH served as reference gene (means ± SD, n = 3 independent experiments with each 4 replicates). *p≤0.05. Ns, not significant.

**Supplemental Table 1.** List of antibodies.

| **Antibodies** | **Application** | **Dosage** | **Source** |
| --- | --- | --- | --- |
| GAPDH | WB | 1:1000 | ZSGB-BIO 17AF0412 |
| GSK-3α | WB | 1:1000 | CST # 4337T |
| GSK-3β | WB | 1:1000 | CST # 12456 |
| Phospho-GSK-3β (Ser9) | WB | 1:1000 | Novus nbp2-67444 |
| Phospho-GSK-3α (Ser21) | WB | 1:1000 | R&D Systems AF4125 |
| Phospho-GSK-3α/β (Tyr216, Tyr279) | WB | 1:1000 | Novus SY26-05 |
| GS | WB | 1:1000 | CST # 3893S |
| Phospho-GS (Ser641) | WB | 1:1000 | CST # 3891 |
| MMP-1 | WB | 1:1000 | CST # 54376 |
| MMP-2 | WB | 1:1000 | CST #40994 |
| MMP-3 | WB | 1:1000 | CST # 14351 |
| MMP-9 | WB | 1:1000 | CST # 13667S |
| LC3 A/B | WB | 1:1000 | CST # 12741 |
| SQSTM1/p62 (D1Q5S) | WB | 1:1000 | CST # 39749S |
| Phospho-p44/42 MAPK (ERK1/2)  (Thr202/Tyr204) | WB | 1:1000 | CST # 4370S |
| p44/42 MAPK (ERK1/2) | WB | 1:1000 | CST # 4695S |
| Goat anti-Mouse IgG (H+L) Secondary Antibody, HRP | WB | 1:3000 | Thermo 31430 |
| Goat anti-Rabbit IgG (H+L) Secondary Antibody, HRP | WB | 1:3000 | Thermo 31460 |
| PKC-δ | WB | 1:1000 | CST # 2058S |
| PKC | WB | 1:1000 | SAB #41347 |
| Phospho-mTOR (Ser2481) | WB | 1:1000 | CST # 2974S |
| mTOR | WB | 1:1000 | CST # 2972S |
| MMP-9 | IHC | 1:500 | Abcam ab38898 |

WB, Western Blot; IHC, Immunohistochemistry.
